# Impact of COVID‐19 lockdown restrictions on hepatitis C testing in Australian primary care services providing care for people who inject drugs

**DOI:** 10.1111/jvh.13723

**Published:** 2022-07-05

**Authors:** Michael W. Traeger, Daniela K. van Santen, Rachel Sacks‐Davis, Jason Asselin, Allison Carter, Joseph S. Doyle, Alisa Pedrana, Anna L. Wilkinson, Jessica Howell, Rebecca Thatcher, John Didlick, Basil Donovan, Rebecca Guy, Margaret E. Hellard, Mark A. Stoové, the Australia Collaboration for Coordinated Enhanced Sentinel Surveillance of Sexually Transmissible Infections and Blood Borne Viruses (ACCESS)

**Affiliations:** ^1^ Burnet Institute Melbourne Victoria Australia; ^2^ School of Public Health and Preventive Medicine Monash University Melbourne Victoria Australia; ^3^ Department of Infectious Disease, Research and Prevention Public Health Service of Amsterdam Amsterdam the Netherlands; ^4^ Kirby Institute UNSW Sydney Sydney New South Wales Australia; ^5^ Faculty of Health Sciences Simon Fraser University Vancouver British Columbia Canada; ^6^ Australian Human Rights Institute UNSW Sydney Sydney New South Wales Australia; ^7^ Department of Infectious Diseases The Alfred and Monash University Melbourne Victoria Australia; ^8^ Department of Gastroenterology St Vincent's Hospital Melbourne Victoria Australia; ^9^ Department of Medicine University of Melbourne Melbourne Victoria Australia; ^10^ Access Health Melbourne Victoria Australia; ^11^ Hepatitis Australia Canberra Australian Capital Territory Australia; ^12^ Sydney Sexual Health Centre Sydney New South Wales Australia; ^13^ Peter Doherty Institute Melbourne Victoria Australia

**Keywords:** COVID‐19, hepatitis C, lockdowns, people who inject drugs

## Abstract

In 2020, the Australian state of Victoria experienced the longest COVID‐19 lockdowns of any jurisdiction, with two lockdowns starting in March and July, respectively. Lockdowns may impact progress towards eliminating hepatitis C through reductions in hepatitis C testing. To examine the impact of lockdowns on hepatitis C testing in Victoria, de‐identified data were extracted from a network of 11 services that specialize in the care of people who inject drugs (PWID). Interrupted time‐series analyses estimated weekly changes in hepatitis C antibody and RNA testing from 1 January 2019 to 14 May 2021 and described temporal changes in testing associated with lockdowns. Interruptions were defined at the weeks corresponding to the start of the first lockdown (week 14) and the start (week 80) and end (week 95) of the second lockdown. Pre‐COVID, an average of 80.6 antibody and 25.7 RNA tests were performed each week. Following the first lockdown in Victoria, there was an immediate drop of 23.2 antibody tests and 8.6 RNA tests per week (equivalent to a 31% and 46% drop, respectively). Following the second lockdown, there was an immediate drop of 17.2 antibody tests and 4.6 RNA tests per week (equivalent to a 26% and 33% drop, respectively). With testing and case finding identified as a key challenge to Australia achieving hepatitis C elimination targets, the cumulative number of testing opportunities missed during lockdowns may prolong efforts to find, diagnose and engage or reengage in care of the remaining population of PWID living with hepatitis C.

**Abbreviations**
ACCESS: Australian Collaboration for Coordinated Enhanced Sentinel Surveillance of Sexually Transmissible Infections and Blood Borne Viruses
COVID‐19: coronavirus disease 2019
DAA: direct‐acting antivirals
HCV: hepatitis C virus
PBS: Pharmaceutical Benefits Scheme
RNA: ribonucleic acid
PWID: people who inject drugs
WHO: World Health Organization

## 1 INTRODUCTION

A key pillar of public health responses to COVID‐19 has been various levels and periods of ‘lockdown’, which have included restrictions on people's movements and the closing of workplaces, services and social venues. While health services have general remained open during these periods, health system pressures associated with COVID‐19^1^ and community concerns about attending health services and COVID‐19 acquisition risk^2^, ^3^, ^4^ have challenged the maintenance of routine health service delivery. Of great concern is the impact of the pandemic and subsequent government‐imposed restrictions on access to healthcare,^5^, ^6^ including testing and treatment for other communicable diseases. Global disease elimination strategies, which necessitate high rates of testing and treatment among priority populations such as those for the elimination of hepatitis B and hepatitis C, are likely to be hindered by widespread reductions in access to healthcare during the COVID‐19 pandemic.

Australia has a longstanding strategic response to hepatitis C and has set national targets that align with global elimination targets set by the WHO that aim to reduce hepatitis C incidence by 90% compared with 2015 levels by 2030.^7^ High coverage of testing among people who inject drugs (PWID) and access to treatment for all are key to Australia's hepatitis C elimination strategy.^8^ While the availability of direct‐acting antiviral (DAA) therapy in 2016 leads to a rapid escalation in testing and case finding, decelerating rates of testing and case‐finding since late 2016 are threatening Australia's HCV elimination progress.^9^ Modelling work shows that without significant and sustained increases in testing among people exposed to HCV, including PWID and other people living with HCV, and subsequent timely referral to care and treatment, Australia will not reach its 2030 elimination goals.^10^ Restrictions implemented in response to COVID‐19 may further impact Australia's progress towards eliminating hepatitis C through reductions in hepatitis C testing.

The first case of COVID‐19 in Australia was diagnosed on 25 January 2020. In response, from March 2020, the Australian federal and state governments introduced restrictions in order to curtail COVID‐19 transmission. Each of Australia's states and territories subsequently implemented varying levels of restrictions based on directions from state‐based health authorities in response to local epidemic characteristics. In addition, telehealth (video‐call) consultations were made available to everyone eligible for Australia's universal healthcare system, Medicare.^11^ While many states and territories experienced a single‐wave epidemic of COVID‐19 in early 2020, a larger second wave of COVID‐19 transmission in the state of Victoria which began in July of 2020 led to 4 months of lockdown across the state.^12^ These lockdown measures in Victoria were widely successful in curtailing COVID‐19 transmission, with the number of daily COVID‐19 cases peaking at 725 and then returning to many months of zero COVID‐19 cases.^13^ Given significant and sustained lockdowns occurring in NSW and Victoria in from June 2021 following the introduction of the COVID‐19 delta variant and significant waves of COVID‐19 transmission,^14^ we used interrupted time‐series analyses to retrospectively examine the impact of the preceding lockdowns during 2020 in Victoria on hepatitis C testing, as well as rates of recovery following these lockdowns, among individuals attending a network of services in Victoria specializing in the care of PWID.

## 2 METHODS

### 2.1 Data source

Clinical data were extracted from a network of 11 general practice and community health clinics in the state of Victoria participating in the Australian Collaboration for Coordinated Enhanced Sentinel Surveillance of Blood Borne Viruses and Sexually Transmissible Infections or ACCESS.^15^ The ACCESS protocol has been published elsewhere.^15^ ACCESS clinics included in this analysis were sentinel surveillance sites that were selected based on high hepatitis C caseloads and provision of services tailored towards PWID, including opioid agonist therapy prescribing and co‐location with needle and syringe programs. Nine clinics were located in the Melbourne metropolitan area and two were in regional Victoria. Patient demographics and hepatitis C antibody and RNA test results were retrospectively extracted using GRHANITE™ data extraction software, which was designed specifically for the secure collection of de‐identified health data.^16^ Using GRHANITE, patient records are linked within and across sites using a highly sensitive algorithm which utilizes non‐identifying probabilistic linkage keys derived from, but not containing, patient identifiers, including patient name, date of birth, sex and Medicare card number.^17^

### 2.2 Outcomes

Using data from all services during the 125 weeks between 1 January 2019 and 25 May 2021, we explored three primary outcomes: (1) weekly number of HCV antibody tests conducted; (2) weekly number of HCV RNA tests conducted; and (3) weekly number of individuals tested for HCV (antibody or RNA) for the first time on record in the ACCESS system (‘first‐time testers’). In order to help contextualize changes in testing, we examined whether similar changes in clinical consultations occurred during the study period by including weekly number of clinical consultations among all patients attending the network of clinics (including in‐person and telehealth consultations) as a secondary outcome.

### 2.3 Timeline of restrictions

During 2020, a range of restrictions issued by Victoria's Chief Health Officer were implemented across Victoria in different stages. Stages 1 and 2 involved limits on public and private gatherings, interstate travel restrictions and capacity limits in restaurants, bars and clubs, as well as at weddings, funerals and religious gatherings. Stage 3 restrictions involved stricter ‘lockdown’ measures, in which people were only allowed to leave their homes for four reasons; getting food and supplies, daily exercise, accessing medical care and caregiving. The first stage 3 restrictions in Victoria were introduced on 30 March 2020 and ceased on 11 May 2020.^18^ Telehealth consultations were made available through Medicare on 13 March 2020, extending until 31 December 2021.^11^ Melbourne returned to a second lockdown on 8 July 2020 which ran until 26 October 2020.^19^, ^20^ In the first half of 2021, in response to new outbreaks, greater Melbourne returned to a 5‐day lockdown from 12 February 2021 to 16 February 2021 and again from 25 May 2021 to 10 June 2021. Given insufficient follow‐up available at time of analysis, we censored our analysis on 24 May 2021, prior to Melbourne's fourth lockdown.

### 2.4 Observation periods

The unit of observation for this time‐series analysis was weekly number of each outcome (tests/consultations) conducted across the network. Week number was defined as each consecutive 7‐day period beginning 1 January 2019–7 January 2019 (week 1) to 19 May 2021–25 May 2021 (week 125). Four observation periods were defined based on week number to align with the implementation and easing of lockdowns; pre‐lockdowns (period 1), first lockdown and post‐first lockdown (period 2), second lockdown (period 3) and post‐lockdowns (period 4).

Using interrupted time‐series analysis, it is recommended to have at least eight time points before and after the interruption in order to have sufficient power to estimate regression coefficients.^21^ Additionally, at least eight time points are required between multiple interruption points in order to estimate their impact independently.^21^ Given the short time between the end of the first lockdown and the start of the second lockdown, we were not able to assess an additional interruption at the end of the first lockdown. As such, the first lockdown period and the period between the end of the first lockdown and start of the second lockdown were considered as a single 14‐week period. The third lockdown in Victoria was a ‘snap‐lockdown’ which lasted only 5 days and as such was not consider as a separate period. See Table S1 for timeline of lockdown restrictions and analysis observation periods.

For each outcome (antibody test, RNA test and consultation), over the entire study period and across each of the four defined observation periods, we calculated: (1) the total number of unique individual with the outcome (i.e. the number tested or the number with a consultation, respectively), (2) the total count of the outcome and (3) the average count of the outcome per week. We calculated the relative reduction in the average number of tests (for each of the testing outcomes) or consultations conducted per week during periods two, three and four compared with during period one (pre‐COVID).

### 2.5 Interrupted time‐series analysis

To estimate trends in HCV testing and the number of consultations across each period, and to explore changes in testing and consultations at the introduction of lockdowns and the easing of the second lockdown, we performed three interrupted time‐series analyses. Analyses were conducted by fitting Prais–Winston linear regression models, which account for autocorrelation between weekly observations.

Three interruptions were chosen to reflect the beginning of each observation period. For each outcome, coefficients estimated from the interrupted time‐series analysis included the pre‐lockdown trend (β1, the estimated weekly mean change in outcome during period 1), the immediate change in outcome level at the start of each period (β2, β4, β6) and the change in slope at the beginning of each period (β3, β5, β7) (Box 1). We also calculated the trend during periods 2–4 with corresponding 95% confidence intervals and *p*‐values. We report the predicted values at each interruption estimated using the trend prior to and after the interruption, respectively, and the relative differences. Analyses were disaggregated by sex.

### 2.6 Ethics

Ethics approval for ACCESS was provided by the Human Research Ethics Committees at Alfred Hospital (248/17), Aboriginal Health and Medical Research Council (1099/15), ACON (2015/14), Victorian AIDS Council/Thorne Harbour Health (VAC REP 15/003), and St. Vincent's Hospital (08/051). As our study analyses de‐identified data collected under the auspices of public health surveillance, individual patient consent was not required. Individuals were able to opt‐out of the surveillance system if they wish.

## 3 RESULTS

### 3.1 Mean number of tests and consultations across each period

Table 1 shows the average number of antibody tests, RNA tests, first‐time HCV testers and consultations per week in period 1 (pre‐lockdown), period 2 (during the first lockdown and prior to the second lockdown), period 3 (during the second lockdown) and period 4 (post‐second lockdown).

**TABLE 1:** Total number of consultations and HCV tests conducted during the pre and post‐COVID study periods across all included services

	Whole study period		Period 1 c, Pre‐lockdown period			Period 2, First/post‐first lockdown period				Period 3, Second lockdown period				Period 4, Post‐lockdowns			
	1 Jan 2019–24 May 2021		1 January 2019–31 March 2020			1 April 2020–7 July 2020				8 July 2020–27 October 2020				28 October 2020–24 May 2021			
Outcome	Outcome total	Number unique individuals	Outcome total	Number unique individuals	Weekly mean	Outcome total	Number unique individuals	Weekly mean	Relative change in weekly mean compared to period 1	Outcome total	Number unique individuals	Weekly mean	Relative change in weekly mean compared to period 1	Outcome total	Number unique individuals	Weekly mean	Relative change in weekly mean compared to period 1
Antibody tests	8748	7812	5237	4925	80.6	817	804	58.4	−28%	817	810	51.1	−37%	1877	1824	62.6	−22%
RNA tests	2403	2001	1673	1521	25.7	160	155	11.4	−56%	187	183	11.7	−54%	383	368	12.8	−50%
First‐time testers	5817	5817	3570	3570	54.9	536	536	38.3	−30%	527	527	32.9	−40%	1184	1184	39.5	−28%
Consultations	685,004	103,341	340,908	74,296	5244.7	78,558	36,959	5611.3	7%	89,669	37,258	5604.3	7%	175,869	53,846	5862.3	12%

#### 3.1.1 Antibody tests

A total of 8748 hepatitis C antibody tests were performed among 7812 individuals during the entire observation period. The mean number of antibody tests performed per week during period 1 was 80.6, which dropped to 58.4 during period 2 (28% less than pre‐lockdown) and to 51.1 during period 3 (37% less than pre‐lockdown). During the period 4, the weekly mean was 62.6 or 22% less than pre‐lockdown.

#### 3.1.2 RNA tests

A total of 2403 hepatitis C RNA tests were performed among 2001 individuals during the entire observation period. The mean number of RNA tests performed per week during period 1 was 25.7, which dropped to 11.4 during period 2 (56% less than pre‐lockdown). During period 3, the weekly mean was 11.7 (54% less compared with pre‐lockdown). During period 4, the weekly mean was 12.8 or 50% less than pre‐lockdown.

#### 3.1.3 First‐time HCV testers

During the entire observation period, 5817 individuals were tested for HCV (antibody or RNA) for the first time recorded in the ACCESS system. The mean number of first‐time HCV testers per week during period 1 was 54.9, which dropped to 38.3 during period 2 (30% less than pre‐lockdown). During period 3, the weekly mean was 32.9 (40% less compared with pre‐lockdown) and during period 4, the weekly mean was 39.5 (28 less than pre‐lockdown).

#### 3.1.4 Consultations

During the entire observation period, there were a total of 685,004 clinical consultations among 103,341 individuals. The mean number of consultations occurring per week during period 1 was 5244.7, which increased to 5611.3 in period 2 (7% more than pre‐lockdown) and to 5604.3 during period 3 (7% more than pre‐lockdown). During period 4, the weekly mean number of consultations was 5862.3 or 12% more than the pre‐lockdown period.

### 3.2 Interrupted time‐series analyses

Table 2 shows regression coefficients for each interrupted time‐series model. Table 3 shows relative drops in testing and consultations associated with the introduction of each lockdown.

**TABLE 2:** Estimated regression coefficients from interrupted time‐series analyses

Interruption			Weekly number of antibody tests			Weekly number of RNA tests			Weekly number of first‐time testers			Weekly number of consultation		
			Coefficient	95% CI	*p*‐value	Coefficient	95% CI	*p*‐value	Coefficient	95% CI	*p*‐value	Coefficient	95% CI	*p*‐value
	Period 1 trend	β1	−0.17	−0.35–0.01	.058	−0.21	−0.35 to −0.07	.003	−0.12	−0.26 to 0.01	.075	2.65	−6.65 to 11.95	.574
First lockdown implemented	Absolute level change at start of first lockdown	Β2	−23.2	−38.4 to −8.1	.003	−8.6	−19.3 to 2.1	.115	−18.3	−30 to −6.6	.002	−91	−857.5 to 675.6	.815
	Period 2 trend		1.03	−0.75 to 2.8	.254	0.27	−0.98 to 1.52	.673	0.89	−0.48 to 2.26	.202	57.22	−32.32 to 146.75	.208
	Difference in trends (Period 2 – Period 1)	Β3	1.2	−0.59 to 2.98	.186	0.48	−0.79 to 1.74	.456	1.01	−0.37 to 2.39	.149	54.57	−35.66 to 144.79	.233
Second lockdown implemented	Absolute level change at start of second lockdown	Β4	−17.2	−37 to 2.5	.087	−4.6	−18 to 8.8	.499	−16.4	−31.6 to −1.2	.035	−421.4	−1401.5 to 558.8	.396
	Period 3 trend		0.3	−1.15 to 1.76	.679	0.34	−0.7 to 1.37	.52	0.61	−0.52 to 1.73	.287	−1.15	−74.89 to 72.58	.975
	Difference in trends (Period 3 – Period 2)	Β5	−0.72	−3.02 to 1.58	.535	0.07	−1.62 to 1.76	.934	−0.28	−2.06 to –1.5	.755	−58.37	−176.71 to 59.97	.331
Second lockdown ended	Absolute level change at end of second lockdown	Β6	6.8	−10.2 to 23.8	.430	−4.00	−15.9 to 7.9	.509	1.8	−11.3 to 14.9	.789	−181.2	−1036.7 to 674.2	.676
	Period 4 trend		0.14	−0.42 to 0.71	.618	0.15	−0.28 to 0.57	.494	−0.04	−0.47 to 0.4	.869	31	1.68–60.32	.038
	Difference in trends (Period 4 – Period 3)	Β7	−0.16	−1.72 to 1.4	.839	−0.19	−1.33 to 0.95	.74	−0.64	−1.85 to 0.56)	.294	32.15	−48.02 to 112.33	.429
	Model fit, R^2^ (AIC)		0.415 (1007.820)			0.3086 (866.825)			0.451 (940.802)			0.0818 (1957.710)		

**TABLE 3:** Predicted values and relative level change at each interruption

	Predicted value at week 66 (period 1 trends)	Predicted value at week 66 (period 2 trend)	% Level change at week 66	Predicted value at week 80 (period 2 trend)	Predicted value at week 80 (period 3 trend)	% Level change at week 80	Predicted value at week 80 (period 3 trends)	Predicted value at week 80 (period 4 trend)	% Level change at week 80
Antibody tests	74.9	51.7	−31%	66.1	48.8	−26	53.7	60.5	13
RNA tests	18.7	10.1	−46%	13.8	9.2	−33	14.6	10.6	−27
First‐time HCV tested	50.8	32.5	−36%	44.9	28.5	−37	38.2	40	5
Consultations	5327.6	5236.6	−2%	6037.6	5616.3	−7	5597.8	5416.6	−3

#### 3.2.1 Antibody tests

**FIGURE 1:**
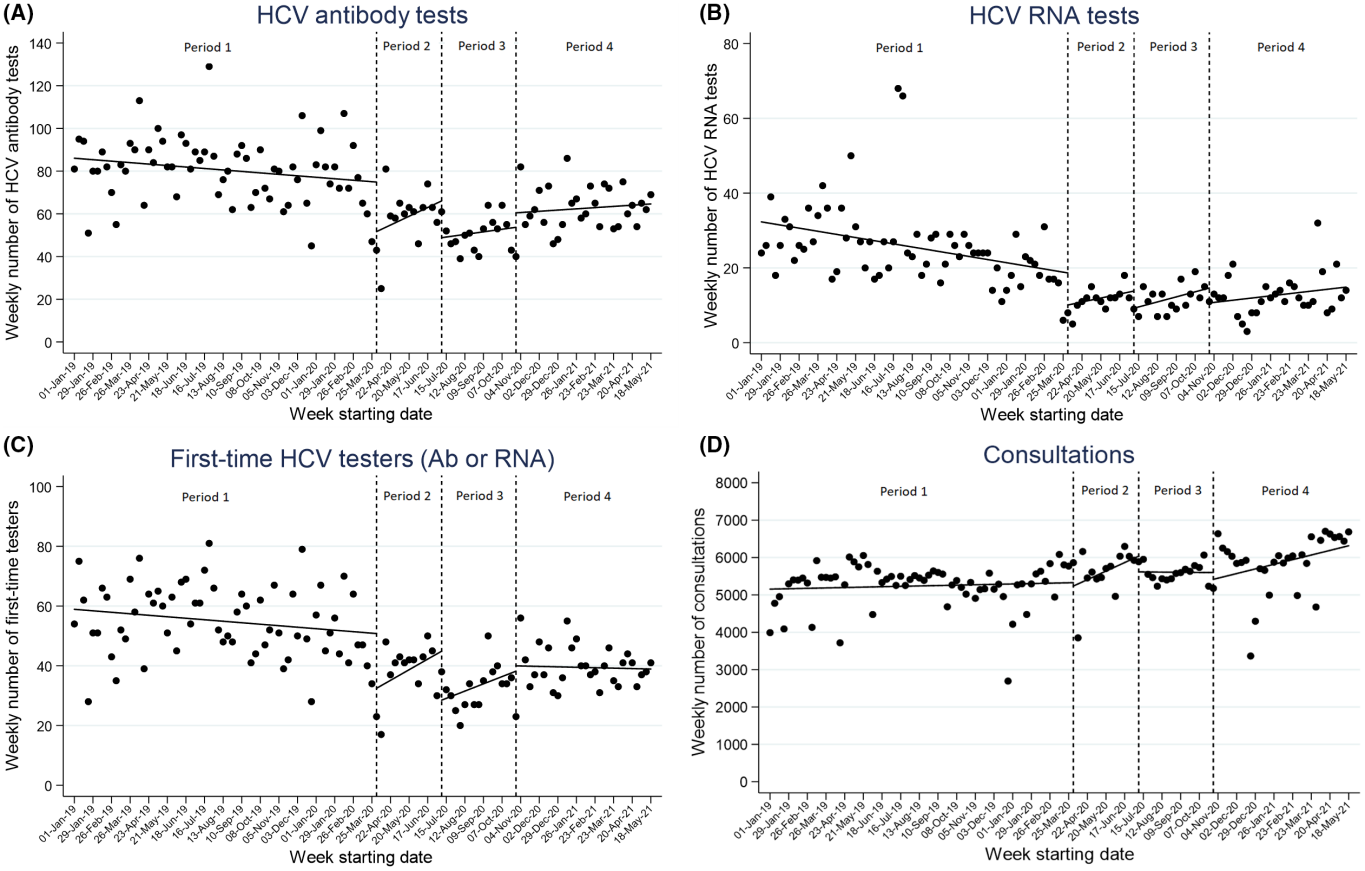
Interrupted time‐series analysis of weekly number of (A) HCV antibody tests, (B) HCV RNA tests, (C) people tested for HCV (Ab or RNA) for the first time on record and (D) clinical consultations, across 11 Victorian services. Dashed lines show interruptions at week 66—start of first lockdown, week 80—start of second lockdown and week 96—end of second lockdown. Dots represent actual weekly number and solid line predicted weekly number

The number of antibody tests performed each week across the network was declining slowly prior to the introduction of the first lockdown (b1 = −0.17, *p* = .058). The introduction of the first lockdown was associated with an immediate absolute drop of 23 antibody tests per week (95% CI = 8–38, *p* = .003), which was equivalent to an immediate relative drop of 31% tests per week. In the period between the introduction of the first and second lockdowns (period 2), antibody testing slightly recovered by an average of 1.0 test per week (*p* = .254). The introduction of the second lockdown was associated with a further absolute drop of 17 antibody tests (95% CI = 3–37, *p* = .087), equivalent to a 26% drop. During the second lockdown (period 3), antibody testing increased by an average of 0.3 tests per week (*p* = .679). The end of the second lockdown was associated with an absolute increase of 6.8 tests per week (95% CI = −10–24, *p* = .430), equivalent to a 13% increase. After the second lockdown (period 4), antibody testing increased by an average of 0.14 tests per week (*p* = .618) to 24 May 2021 (Figure 1A). The declining trend in antibody testing pre‐COVID was more pronounced among males; however, similar relative drops at the introduction of lockdowns were observed for males and females (Table S2).

#### 3.2.2 RNA tests

The number of RNA tests performed across the network each week was declining prior to the introduction of the first lockdown (b1 = −0.21, *p* = .003). The introduction of the first lockdown was associated with an immediate absolute drop in weekly number of RNA tests performed of 8.6 (95% CI = −2–19, *p* = .113), equivalent to a relative reduction of 46%. In the period between the introduction of the first and second lockdowns (period 2), RNA testing increased by an average of 0.27 tests per week (*p* = .673) followed by a further absolute drop of 4.6 RNA tests per week (95% CI = −8 to 18, *p* = .499) at the start of the second lockdown, equivalent to a 33% drop. During the second lockdown (period 3), RNA testing increased at an average of 0.34 tests per week (*p* = .494). The end of the second lockdown was associated with an absolute drop of 4.0 tests per week (95% CI = −15 to 8, *p* = .509), equivalent to a 27% decrease. After the second lockdown (period 4), RNA testing increased by an average of 0.15 tests per week (*p* = .494) to 24 May 2021 (Figure 1B). Similar trends in RNA testing were observed for males and females, with a higher relative drop in RNA testing at the start of lockdown 2 for females, although the mean number of RNA tests being performed just prior to lockdown 2 was low for females (4.1 per week) (Table S4).

#### 3.2.3 First‐time HCV testers

The number of individuals tested for HCV for the first time on record each week across the network was declining slowly prior to the introduction of the first lockdown (b1 = −0.12, *p* = .075). The introduction of the first was associated with an immediate absolute drop of 18 first‐time testers per week (95% CI = 7–30, *p* = .002), which was equivalent to an immediate relative drop of 36%. In the period between the introduction of the first and second lockdowns (period 2), the weekly number of first‐time testers recovered by an average of 0.9 person per week (*p* = .202). The introduction of the second lockdown was associated with a further absolute drop of 16 first‐time testers (95% CI = 1–32, *p* = .035), equivalent to a 37% drop. During the second lockdown (period 3), the weekly number of first‐time testers increased by an average of 0.6 per week (*p* = .287). The end of the second lockdown was associated with an absolute increase of 1.8 first‐time testers per week (95% CI = −15–11, *p* = .789) equivalent to a 5% increase. After the second lockdown (period 4), the weekly number of first‐time testers was stable (*p* = .869) to 24 May 2021 (Figure 1C). Similar to antibody testing, the declining trend in antibody testing pre‐COVID was more pronounced among males; however, similar relative drops at the introduction of lockdowns were observed for males and females (Table S6).

#### 3.2.4 Consultations

The number of consultations among all patients across the network each week was stable prior to the introduction of the first lockdown (b1 = 2.65, *p* = .574). The introduction of the first lockdown at week 66 was associated with an immediate absolute drop of 91 consultations (95% CI = −675.6–857.5, *p* = .815), equivalent to a 1.7% relative drop. In the period between the introduction of the first and second lockdowns (period 2), the number of weekly consultations increased by an average of 57.2 per week (*p* = .252). The introduction of the second lockdown was associated with an absolute drop of 421 consultations per week (95% CI = −558.8 to 1401.5, *p* = .396), equivalent to a 7% drop. During the second lockdown (period 3), consultations were stable (−1.2/week, *p* = .975). The end of the second lockdown was associated with an absolute drop of 181 consultations per week (95% CI = −674.2 to 1036.7, *p* = .676), equivalent to a 3.2% decrease. After the second lockdown (period 4), consultations increased by an average of 31 per week (*p* = .038) to 24 May 2021 (Figure 1D).

## 4 DISCUSSION

Across this network of sentinel clinics specializing in the care of PWID, moderate drops in hepatitis C antibody and RNA testing were observed following the introduction of each COVID‐19‐related lockdown. Testing was slow to recover during lockdown periods and after restrictions were lifted. During periods of COVID‐19‐related lockdowns, antibody testing was 28%–37% lower than during the 65 weeks prior to the first lockdown in March 2020. While we observed some recovery in antibody testing in the months following the lockdowns, the average level of antibody testing had not returned to pre‐COVID levels by the end of May 2021, and the number of individuals being tested for HCV for the first time did not change in the months following lockdowns. Despite drops in hepatitis C testing, the number of consultations across the network was slightly higher during and post‐lockdown periods, likely indicative of the utilization of telehealth consultations.

A crucial element of Australia's elimination strategy relies on active case finding of undiagnosed hepatitis C. In the context of the maturing hepatitis C epidemic in Australia, increased and more broad‐based antibody testing is required to find remaining cases in the community. Modelling work suggests that even though increased HCV antibody testing will result in an overall lower test yield (proportion of tests performed which return positive results), achieving hepatitis C elimination targets will be difficult without a substantial increase in anti‐HCV antibody testing.^10^ Previous analysis of data from Victorian ACCESS clinics showed that antibody test positivity has remained relatively stable from 2013 to 2019 at around 10%.^22^ Strategies should aim to maintain broad‐based and frequent testing in services attended by PWID, including identifying those who have not been tested for hepatitis C recently and retesting those with any ongoing risk behaviour. Programmes which provide financial incentives for undergoing hepatitis C testing may be effective in engaging new and returning PWID at primary care services.^23^ However, as the number of individuals who have been previously treated for hepatitis C increases, detection of new cases will increasingly rely on RNA testing.

The introduction of the first lockdown restrictions in Victoria was associated with an immediate 46% drop in RNA testing, with slow recovery in RNA testing consistent across subsequent lockdown and post‐lockdown periods. When compared with the pre‐COVID period, RNA testing more than halved during the lockdown and post‐lockdown periods. In contrast to antibody testing, there was no absolute increase in RNA testing detected at the end of the second lockdown. Declining trends in RNA testing prior to COVID‐19 likely reflect steady declines in treatment commencements and associated diagnostic testing since 2017.^24^, ^25^ It is estimated that by the end of 2020, there were still 117,800 people living in Australia with hepatitis C yet to be treated.^26^ There are concerns that COVID‐19 may have further reduced treatment opportunities and substantially impacted hepatitis C elimination efforts. The number of DAA prescriptions dispensed through the PBS in 2020 was 8099, down from the 11,314 recorded in 2019),^26^ which is now well below an estimated minimum of 13,680 annual treatments needed to achieve elimination targets.^27^ Although this drop in national DAA prescribing is consistent with annual drops over previous years, larger declines in DAA treatments in 2020 compared with previous years have been reported in Victoria (28% drop in DAA treatments in 2020 compared with 18% drop in 2019).^28^ Despite slow recoveries in testing into 2021, the cumulative weekly reductions in testing during the COVID‐19 lockdowns may have implications for HCV‐related liver disease and transmission through missed diagnoses, less liver screening and delayed treatment of those with hepatitis C.

Across our network, the relative reduction in testing during lockdown periods compared with pre‐COVID levels was in contrast with changes in the number of consultations, which increased by 7%. Greater drops in antibody testing relative to clinic consultations may reflect greater reductions in attendance among people at risk for hepatitis C compared with those seeking general care, a reduction in the level of risk behaviour associated with hepatitis C risk, priorities within the clinics shifting away from hepatitis C screening during periods of COVID‐19 transmission or competing priorities among individuals within the community. The observed increase in consultations is likely due to clinics switching to providing telehealth consultations, as reimbursements paid to GPs increased following COVID‐19, with reimbursements greater for telehealth compared with face‐to‐face consultations.^11^ Analysis of Medicare claims data shows increases in telehealth consultations in general practice following COVID‐19 lockdowns in Australia,^29^ with one study finding that 68% of doctors reported all or most of their consultations had moved to telephone or video in June 2020.^30^ It is also possible that the increase of consultations was partly driven by consultations associated with COVID‐19 testing, although the majority of COVID‐19 testing was done at state‐run testing hubs. While telehealth consultations may be conducive to general healthcare, they likely present additional barriers to diagnostic testing visits which require laboratory testing and an associated follow‐up visit. Many individuals have reported hesitance to leave home during periods of COVID transmission in fear of contracting the illness.^31^ These barriers may be magnified among PWID, who face an increased risk of serious illness from COVID‐19 given the high prevalence of chronic medical conditions among this population.^32^ Further, COVID‐19‐related social and physical restrictions have the potential to disproportionately affect PWID, who may experience disproportionately higher rates of job loss and compounding forms of stigma during the pandemic, as well as reduced access to harm reduction and mental health services.^32^

While trends in HCV testing post‐lockdowns were observed to be heading towards pre‐lockdown levels, the cumulative number of missed testing opportunities during the lockdown periods may have salient implications for progress towards elimination of hepatitis C. Individuals who may have presented for testing during these periods, however, did not because of restrictions, may not necessarily be represented among those returning for testing after the lockdowns. Our observation that consultations (including telehealth consultations) did not decline in line with in testing levels during periods of lockdown suggests that many individuals remained engaged in routine care. However, it is likely that the increased burden of other health issues, including mental health, domestic violence, substance dependence and other harms, influenced the priorities of both clients and healthcare providers, contributing to the observed drop in hepatitis C testing. Of note, the drop in first‐time HCV testers at the start of the second lockdown (37%) was greater than the drop in antibody testing (26%) and RNA testing (33%) at the same time, highlighting the potential impact of the restrictions on engaging new patients in HCV testing. Efforts to both engage and re‐engage clients in hepatitis C testing post‐COVID will be crucial.

Ongoing transmission of COVID‐19 globally suggests that COVID‐19‐related disruptions in Australia will likely continue to have a significant impact on the provision of healthcare for years to come. While balancing COVID‐19 response efforts with other health‐related priorities such as hepatitis C elimination may be difficult, maintaining efforts towards elimination targets will be beneficial in the long term. The longer countries take to reach elimination, the less cost‐effective elimination strategies become.^33^ Keeping governments and clinicians engaged in hepatitis C elimination during and post‐COVID will be essential in reaching 2030 elimination targets.

The full impact of COVID‐19 lockdowns on HCV transmission within the community is not yet known. While notification data show declines in hepatitis C diagnoses during the COVID‐19 era,^34^ notification trends are likely influenced by drops in testing. Sentinel surveillance data, such as that collected by ACCESS, will play an important role in monitoring and estimating the effect of COVID‐19 on hepatitis C incidence and in guiding strategies to promote a return to service engagement.

There are several limitations to our study. First, given that these data are de‐identified prior to extraction from routine clinical and laboratory records, we were not able to disaggregate PWID explicitly from other individuals in the data set. Second, given the low number of weeks (time points) between the introduction of the first and second lockdown periods (period 2), we may not have been able to detect trends which did not reach statistical significance. Third, as we were unable to disaggregate telehealth consultations and face‐to‐face consultations, or disaggregate clinical consultations related to HCV care from general health consultations, we could not explore the impact of COVID‐19 on HCV‐related consultations directly.

Across this network of primary care clinics in Victoria, the implementation of state‐wide lockdowns in response to COVID‐19 during 2020 was associated with modest reductions in hepatitis C antibody and RNA testing. While some recovery in hepatitis C testing rates was observed in 2021, the cumulative number of testing opportunities missed during lockdowns may prolong efforts to find and diagnose the remaining population of PWID living with undiagnosed hepatitis C.

## CONFLICT OF INTEREST

MWT has received speaker's fees and investigator‐initiated funding from Gilead Sciences. JSD declares payments to his institution for investigator‐initiated research from AbbVie and Gilead and consultancies from AbbVie, Gilead and Merck. AP declares investigator‐initiated research from AbbVie, Gilead, Merck and consultancies fees from Gilead. JH declares investigator‐initiated funding from Gilead Sciences and Eisai and advisory board fees from Gilead Sciences. MEH received funding for investigator‐initiated research from Gilead Sciences and Abbvie. All other authors declare no conflicts of interest. ACCESS is funded by the Australian Department of Health.

BOX 1 **Model equation and parameters**

**Table:** 

*Y*_*t*_ = *β*_0_ + *β*_1_T_t_ + *β*_2_X_t_ + *β*_3_X_t_T_t_ + *β*_4_Z_t_ + *β*_5_Z_t_T_t_ + *β*_6_A_t_ + *β*_7_A_t_T_t_ + *ε*_t_
*T* = Time (week)
X = 0 prior to first lockdown, X = 1 after introduction of first lockdown
Z = 0 prior to second lockdown, Z = 1 after introduction of second lockdown
A = 0 prior to end of second lockdown, A = 1 after end of second lockdown
Β0 = Intercept (predicted weekly count at Week 1: 1–7 January 2019)
β1 = Period 1 trend (estimated weekly change in outcome during the pre‐lockdown period)
β2 = Level change at start of first lockdown
β3 = Difference between period 2 and period 1 trends
β4 = Level change at start of second lockdown
β5 = Difference between period 3 and period 2 trends
β6 = Level change at end of second lockdown
β7 = Difference between period 4 and period 3 trends

## Supporting information


**Appendix S1** Supplementary Information

## Data Availability

The data that support the findings of this study are available on request from the corresponding author. The data are not publicly available due to privacy or ethical restrictions.
