# Supplementary material for: Impact of COVID‐19 lockdown restrictions on hepatitis C testing in Australian primary care services providing care for people who inject drugs
Source: J Viral Hepat. 2022 Jul 5:10.1111/jvh.13723. Online ahead of print. doi: 10.1111/jvh.13723 (PMC9350356; doi:10.1111/jvh.13723)
Supplement: Supplementary file 1 — Appendix S1 Supplementary Information [file JVH-9999-0-s001.docx]

**SUPLLEMENTARY MATERIALS**

**Impact of COVID-19 lockdown restrictions on hepatitis C testing in Australian primary care services providing care for people who inject drugs**

Michael W. Traeger, Daniela K. van Santen, Rachel Sacks-Davis, Jason Asselin, Allison Carter, Joseph S. Doyle, Alisa Pedrana, Anna L. Wilkinson, Jessica Howell, Rebecca Thatcher, John Didlick, Basil Donovan, Rebecca Guy, Margaret E. Hellard, Mark A Stoové

**Contents**

[Table 1. Timeline of COVID-19 lockdown restrictions in Victoria, and definition of time-series analysis periods 2](#_Toc99658607)

[Antibody testing by Sex 3](#_Toc99658608)

[Table 2: ITSA regression coefficients for weekly antibody tests, by sex 3](#_Toc99658609)

[Table 3: Predicted values and relative change at each interruption for weekly antibody tests, by sex 3](#_Toc99658610)

[Figure 1: Interrupted time-series analysis for weekly number of antibody tests among males. 4](#_Toc99658611)

[Figure 2: Interrupted time-series analysis for weekly number of antibody tests among females. 4](#_Toc99658612)

[RNA testing by Sex 5](#_Toc99658613)

[Table 4: ITSA regression coefficients for weekly RNA tests, by sex 5](#_Toc99658614)

[Table 5: Predicted values and relative change at each interruption for RNA antibody tests, by sex 5](#_Toc99658615)

[Figure 3: Interrupted time-series analysis for weekly number of RNA tests among males. 6](#_Toc99658616)

[Figure 4: Interrupted time-series analysis for weekly number of RNA tests among females. 6](#_Toc99658617)

[First-time HCV testers by Sex 7](#_Toc99658618)

[Table 6: ITSA regression coefficients for weekly number of people tested for HCV (Ab or RNA) for the first time, by sex 7](#_Toc99658619)

[Table 7: Predicted values and relative change at each interruption for people tested for HCV (Ab or RNA) for the first time, by sex 7](#_Toc99658620)

[Figure 5: Interrupted time-series analysis for weekly number first-time HCV testers (males). 8](#_Toc99658621)

[Figure 6: Interrupted time-series analysis for weekly number first-time HCV testers (females). 8](#_Toc99658622)

[Consultations by Sex 9](#_Toc99658623)

[Table 8: ITSA regression coefficients for weekly consultations, by sex 9](#_Toc99658624)

[Table 9: Predicted values and relative change at each interruption for consultations, by sex 9](#_Toc99658625)

[Figure 7: Interrupted time-series analysis for consultations among males. 10](#_Toc99658626)

[Figure 8: Interrupted time-series analysis for consultations among females. 10](#_Toc99658627)

## Table 1. Timeline of COVID-19 lockdown restrictions in Victoria, and definition of time-series analysis periods

| Time-series analysis | | | Public health orders | |
| --- | --- | --- | --- | --- |
| Observation period | Week range | Date range | Description | Date range |
| **Period 1** Pre-Lockdown | Week 1 – Week 65 (65 weeks) | 01-Jan-19 to 31- Mar-20 |  |  |
|  |  |  |  |  |
| **Period 2** First lockdown and post-first lockdown | Week 66 – Week 79  (14 weeks) | 1-Apr-20 to 7-Jul-20 | First lockdown | 30-Mar-20 to 11-May-20 |
|  |  |  |  |  |
|  |  |  |  |  |
|  |  |  |  |  |
| **Period 3** Second lockdown | Week 80 – Week 95  (16 weeks) | 8-Jul-20 to 27-Oct-20 | Second Lockdown | 08-Jul-20 to 26-Oct-20 |
|  |  |  |  |  |
| **Period 4** Post-lockdowns* | Week 96 – Week 125  (30 weeks) | 28-Oct-20 to 24-May-21 |  |  |
|  |  |  |  |  |
|  |  |  | Third Lockdown* | 12-Feb-21 to 16-Feb-21 |
|  |  |  |  |  |
|  |  |  |  |  |
|  |  |  |  |  |

* As Victoria's third lockdown only lasted 5 days, it was not considered as a separate period in the time-series analysis.

# Antibody testing by Sex

## Table 2: ITSA regression coefficients for weekly antibody tests, by sex

|  |  | | Males | | | Females | | |
| --- | --- | --- | --- | --- | --- | --- | --- | --- |
| Interruption |  |  | Coefficient | 95% CI | P-value | Coefficient | 95% CI | P-value |
|  | Period 1 trend | β1 | -0.16 | -0.28 – -0.04 | p=0.008 | -0.01 | -0.11 – 0.08 | p=0.801 |
| 1^st^ lockdown implemented | Absolute level change at start of first lockdown | Β2 | -10.6 | -20.5 – -0.7 | p=0.037 | -12.80 | -20.9 – -4.6 | p=0.003 |
|  | Difference in trends (Period 2 – Period 1) | Β3 | 0.61 | -0.56 – 1.77 | p=0.304 | 0.63 | -0.33 – 1.6 | p=0.198 |
|  | Period 2 trend |  | 0.45 | -0.71 – 1.61 | p=0.447 | 0.62 | -0.34 – 1.58 | p=0.204 |
| 2^nd^ lockdown implemented | Absolute level change at start of second lockdown | Β4 | -7.60 | -20.4 – 5.2 | p=0.239 | -10.50 | -21.3 – 0.3 | p=0.057 |
|  | Difference in trends (Period 3 – Period 2) | Β5 | -0.12 | -1.64 – 1.39 | p=0.871 | -0.56 | -1.79 – 0.66 | p=0.365 |
|  | Period 3 trend |  | 0.32 | -0.63 – 1.27 | p=0.503 | 0.06 | -0.73 – 0.84 | p=0.887 |
| 2^nd^ lockdown ended | Absolute level change at end of second lockdown | Β6 | 0.80 | -10.2 – 11.9 | p=0.882 | 5.10 | -4.1 – 14.3 | p=0.275 |
|  | Difference in trends (Period 4 – Period 3) | Β7 | -0.27 | -1.3 – 0.76 | p=0.600 | 0.04 | -0.79 – 0.88 | p=0.918 |
|  | Period 4 trend |  | 0.05 | -0.33 – 0.42 | p=0.794 | 0.10 | -0.2 – 0.4 | p=0.514 |
|  | Model fit, R^2^ (AIC) |  | 0.373 (885.014) |  |  | 0.378 (889.303) |  |  |

## Table 3: Predicted values and relative change at each interruption for weekly antibody tests, by sex

|  | Predicted value at week 66 (period 1 trends) | Predicted value at week 66 (period 2 trend) | % Level change at week 66 | Predicted value at week 80 (period 2 trend) | Predicted value at week 80 (period 3 trend) | % Level change at week 80 | Predicted value at week 80 (period 3 trends) | Predicted value at week 80 (period 4 trend) | % Level change at week 80 |
| --- | --- | --- | --- | --- | --- | --- | --- | --- | --- |
| Male | 30.7 | 20.1 | -34% | 26.4 | 18.8 | -29% | 23.9 | 24.8 | 3% |
| Female | 44.1 | 31.4 | -29% | 40.1 | 29.6 | -26% | 30.5 | 35.5 | 17% |

## Figure 1: Interrupted time-series analysis for weekly number of antibody tests among males.

## Figure 2: Interrupted time-series analysis for weekly number of antibody tests among females.

# RNA testing by Sex

## Table 4: ITSA regression coefficients for weekly RNA tests, by sex

|  |  | | Males | | | Females | | |
| --- | --- | --- | --- | --- | --- | --- | --- | --- |
| Interruption |  |  | Coefficient | 95% CI | P-value | Coefficient | 95% CI | P-value |
|  | Period 1 trend | β1 | -0.14 | -0.25 – -0.04 | p=0.008 | -0.06 | -0.1 – -0.02 | p=0.002 |
| 1^st^ lockdown implemented | Absolute level change at start of first lockdown | Β2 | -6 | -14.3 – 2.3 | p=0.155 | -3.4 | -6.7 – 0 | p=0.048 |
|  | Difference in trends (Period 2 – Period 1) |  | 0.4 | -0.58 – 1.37 | p=0.425 | 0.16 | -0.24 – 0.55 | p=0.433 |
|  | Period 2 trend | Β3 | 0.25 | -0.72 – 1.22 | p=0.608 | 0.09 | -0.3 – 0.48 | p=0.637 |
| 2^nd^ lockdown implemented | Absolute level change at start of second lockdown | Β4 | -2.6 | -13 – 7.7 | p=0.615 | -2.2 | -6.8 – 2.3 | p=0.334 |
|  | Difference in trends (Period 3 – Period 2) |  | -0.08 | -1.39 – 1.23 | p=0.902 | 0.07 | -0.45 – 0.59 | p=0.792 |
|  | Period 3 trend | Β5 | 0.17 | -0.63 – 0.97 | p=0.676 | 0.16 | -0.18 – 0.51 | p=0.347 |
| 2^nd^ lockdown ended | Absolute level change at end of second lockdown | Β6 | -3.3 | -12.5 – 5.9 | p=0.481 | -0.7 | -4.5 – 3.2 | p=0.736 |
|  | Difference in trends (Period 4 – Period 3) |  | -0.05 | -0.93 – 0.84 | p=0.918 | -0.15 | -0.51 – 0.22 | p=0.433 |
|  | Period 4 trend | Β7 | 0.12 | -0.2 – 0.45 | p=0.455 | 0.02 | -0.11 – 0.14 | p=0.778 |
|  | Model fit, R^2^ (AIC) |  |  |  |  |  |  |  |

## Table 5: Predicted values and relative change at each interruption for RNA antibody tests, by sex

|  | Predicted value at week 66 (period 1 trends) | Predicted value at week 66 (period 2 trend) | % Level change at week 66 | Predicted value at week 80 (period 2 trend) | Predicted value at week 80 (period 3 trend) | % Level change at week 80 | Predicted value at week 80 (period 3 trends) | Predicted value at week 80 (period 4 trend) | % Level change at week 80 |
| --- | --- | --- | --- | --- | --- | --- | --- | --- | --- |
| Male | 12.7 | 6.7 | -47% | 10.2 | 7.6 | -26% | 10.3 | 7 | -32% |
| Female | 6.1 | 2.8 | -55% | 4.1 | 1.8 | -55% | 4.5 | 3.8 | -15% |

## Figure 3: Interrupted time-series analysis for weekly number of RNA tests among males.

## Figure 4: Interrupted time-series analysis for weekly number of RNA tests among females.

# First-time HCV testers by Sex

## Table 6: ITSA regression coefficients for weekly number of people tested for HCV (Ab or RNA) for the first time, by sex

|  |  | | Males | | | Females | | |
| --- | --- | --- | --- | --- | --- | --- | --- | --- |
| Interruption |  |  | Coefficient | 95% CI | P-value | Coefficient | 95% CI | P-value |
|  | Period 1 trend | β1 | -0.11 | -0.2 – -0.02 | p=0.019 | -0.02 | -0.1 – 0.06 | p=0.648 |
| 1^st^ lockdown implemented | Absolute level change at start of first lockdown | Β2 | -7.6 | -15.1 – 0 | p=0.05 | -10.6 | -17.3 – -3.9 | p=0.002 |
|  | Difference in trends (Period 2 – Period 1) |  | 0.44 | -0.45 – 1.33 | p=0.325 | 0.56 | -0.22 – 1.35 | p=0.16 |
|  | Period 2 trend | Β3 | 0.33 | -0.55 – 1.22 | p=0.456 | 0.54 | -0.24 – 1.33 | p=0.171 |
| 2^nd^ lockdown implemented | Absolute level change at start of second lockdown | Β4 | -7 | -16.7 – 2.7 | p=0.157 | -9.7 | -18.5 – -0.9 | p=0.031 |
|  | Difference in trends (Period 3 – Period 2) |  | 0.12 | -1.04 – 1.29 | p=0.832 | -0.35 | -1.36 – 0.66 | p=0.493 |
|  | Period 3 trend | Β5 | 0.46 | -0.27 – 1.19 | p=0.213 | 0.2 | -0.44 – 0.84 | p=0.546 |
| 2^nd^ lockdown ended | Absolute level change at end of second lockdown | Β6 | -3 | -11.4 – 5.5 | p=0.489 | 3.9 | -3.6 – 11.4 | p=0.301 |
|  | Difference in trends (Period 4 – Period 3) |  | -0.45 | -1.24 – 0.33 | p=0.255 | -0.22 | -0.91 – 0.46 | p=0.523 |
|  | Period 4 trend | Β7 | 0 | -0.28 – 0.29 | p=0.978 | -0.03 | -0.27 – 0.22 | p=0.84 |
|  | Model fit, R^2^ (AIC) |  | 0.358 (809.1) |  |  | 0.377 (823.5) |  |  |

## Table 7: Predicted values and relative change at each interruption for people tested for HCV (Ab or RNA) for the first time, by sex

|  | Predicted value at week 66 (period 1 trends) | Predicted value at week 66 (period 2 trend) | % Level change at week 66 | Predicted value at week 80 (period 2 trend) | Predicted value at week 80 (period 3 trend) | % Level change at week 80 | Predicted value at week 80 (period 3 trends) | Predicted value at week 80 (period 4 trend) | % Level change at week 80 |
| --- | --- | --- | --- | --- | --- | --- | --- | --- | --- |
| Male | 21.7 | 14.1 | -35% | 18.8 | 11.8 | -37% | 19.2 | 16.2 | -15% |
| Female | 29 | 18.4 | -36% | 26.1 | 16.4 | -37% | 19.5 | 23.5 | 20% |

## Figure 5: Interrupted time-series analysis for weekly number first-time HCV testers (males).

## Figure 6: Interrupted time-series analysis for weekly number first-time HCV testers (females).

# Consultations by Sex

## Table 8: ITSA regression coefficients for weekly consultations, by sex

|  |  | | Males | | | Females | | |
| --- | --- | --- | --- | --- | --- | --- | --- | --- |
| Interruption |  |  | Coefficient | 95% CI | P-value | Coefficient | 95% CI | P-value |
|  | Period 1 trend | β1 | 1.76 | -2.84 – 6.35 | p=0.45 | 0.88 | -3.88 – 5.65 | p=0.714 |
| 1^st^ lockdown implemented | Absolute level change at start of first lockdown | Β2 | -31.5 | -409.1 – 346.1 | p=0.869 | -59.6 | -453.3 – 334 | p=0.765 |
|  | Difference in trends (Period 2 – Period 1) |  | 24.64 | -19.8 – 69.09 | p=0.274 | 30.01 | -16.33 – 76.34 | p=0.202 |
|  | Period 2 trend | Β3 | 26.4 | -17.7 – 70.5 | p=0.238 | 30.89 | -15.1 – 76.88 | p=0.186 |
| 2^nd^ lockdown implemented | Absolute level change at start of second lockdown | Β4 | -185.6 | -667.9 – 296.6 | p=0.447 | -237.2 | -741.6 – 267.1 | p=0.353 |
|  | Difference in trends (Period 3 – Period 2) |  | -27.89 | -86.25 – 30.47 | p=0.346 | -30.48 | -91.14 – 30.18 | p=0.322 |
|  | Period 3 trend | Β5 | -1.49 | -37.82 – 34.84 | p=0.935 | 0.41 | -37.44 – 38.26 | p=0.983 |
| 2^nd^ lockdown ended | Absolute level change at end of second lockdown | Β6 | -85.5 | -506.8 – 335.9 | p=0.689 | -96.3 | -535.7 – 343.2 | p=0.665 |
|  | Difference in trends (Period 4 – Period 3) |  | 13.87 | -25.67 – 53.4 | p=0.489 | 18.21 | -22.9 – 59.31 | p=0.382 |
|  | Period 4 trend | Β7 | 12.38 | -2.09 – 26.84 | p=0.093 | 18.62 | 3.6 – 33.64 | p=0.016 |
|  | Model fit, R^2^ (AIC) |  | 0.112 (1779.14) |  |  | 0.157 (1793.73) |  |  |

## Table 9: Predicted values and relative change at each interruption for consultations, by sex

|  | Predicted value at week 66 (period 1 trends) | Predicted value at week 66 (period 2 trend) | % Level change at week 66 | Predicted value at week 80 (period 2 trend) | Predicted value at week 80 (period 3 trend) | % Level change at week 80 | Predicted value at week 80 (period 3 trends) | Predicted value at week 80 (period 4 trend) | % Level change at week 80 |
| --- | --- | --- | --- | --- | --- | --- | --- | --- | --- |
| Male | 2601.7 | 2603.5 | 0% | 2941.6 | 2756 | -6% | 2732.1 | 2646.7 | -3% |
| Female | 2723.9 | 2664.3 | -2% | 3096.8 | 2859.6 | -8% | 2866.2 | 2769.9 | -3% |

## Figure 7: Interrupted time-series analysis for consultations among males.

## Figure 8: Interrupted time-series analysis for consultations among females.
